# Supplementary material for: Identification of photoperiod-induced specific miRNAs in the adrenal glands of Sunite sheep (Ovis aries)
Source: Front Vet Sci. 2022 Jul 22;9:888207. doi: 10.3389/fvets.2022.888207 (PMC9354845; doi:10.3389/fvets.2022.888207)
Supplement: Supplementary file 1 [file Data_Sheet_1.ZIP › Supplementary file/miRDeep2 file.zip!/miRDeep2 file/Know_Structure/SPLP42A2/oar-mir-655.pdf]

miRBase precursor : oar-mir-655
Total read count : 2615
oar-miR-655-5p read count : 8
oar-miR-655-3p read count : 2607
remaining reads : 0

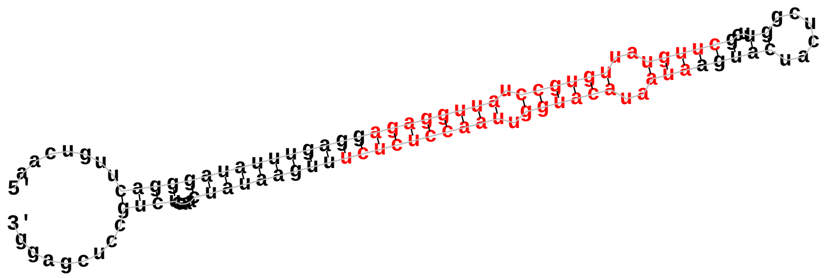

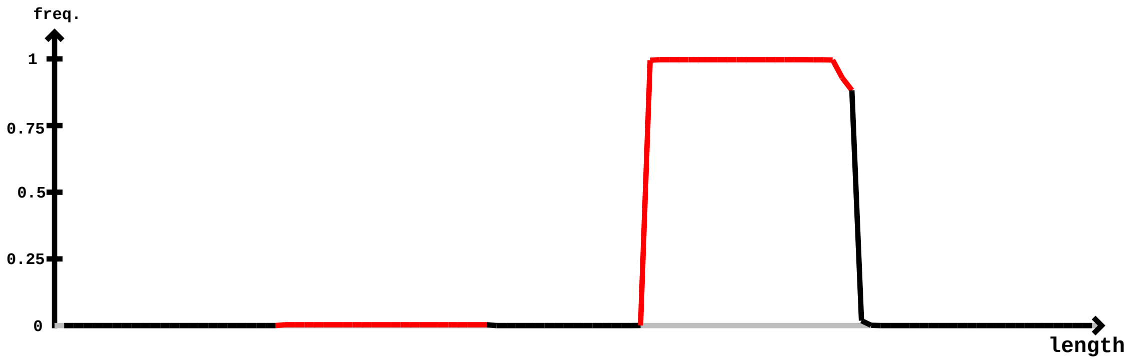

oar-miR-655-5p

oar-miR-655-3p

```
5'- aacuguucagggauauuugaggagagguuauccguguuauguucgcuuggcucaucaugaauaauacaugguuaaccucucuuugaauaucaaacucugccucgagg   -3'   exp
    ...........(((((((((((((((((((.((((((..((((((..((......))))))))..)))))).)))))))))))))))))))...(((......))).     reads    mm    sample
    ......................agagguuauccguguuauguuc...............................................................     8        0     seq
    ...........................................................aauaauacaugguuaaccucu...........................     1        0     seq
    ............................................................auaauacaugguuaacc..............................     1        0     seq
    ............................................................auaauacaugguuaaccuc............................     2        0     seq
    ............................................................auaauacaugguuaaccucu...........................     174      0     seq
    ............................................................auaauacaugguuaaccucuc..........................     122      0     seq
    ............................................................auaauacaugguuaaccucucu.........................     2256     0     seq
    ............................................................auaauacaugguuaaccucucuu........................     44       0     seq
    ............................................................auaauacaugguuaaccucucuuu.......................     2        0     seq
    .............................................................uaauacaugguuaaccucucu.........................     3        0     seq
    .............................................................uaauacaugguuaaccucucuu........................     1        0     seq
    .............................................................uaauacaugguuaaccucucuuu.......................     1        0     seq
```
